# Competitive success of southern populations of *Betula pendula* and *Sorbus aucuparia* under simulated southern climate experiment in the subarctic

**DOI:** 10.1002/ece3.3026

**Published:** 2017-05-30

**Authors:** Kari Taulavuori, Erja Taulavuori, Karita Saravesi, Tanja Jylänki, Aila Kainulainen, Jonna Pajala, Annamari Markkola, Otso Suominen, Kari Saikkonen

**Affiliations:** ^1^ Department of Ecology and GeneticsUniversity of OuluOuluFinland; ^2^ Centre for Environmental ResearchKevo Subarctic Research InstituteUniversity of TurkuTurkuFinland; ^3^ Natural Resources Institute Finland (Luke)TurkuFinland

**Keywords:** autumn coloration, *Betula pendula*, common garden, light environment, local adaptation, shoot elongation, *Sorbus aucuparia*, subarctic, transplantation, warming climate

## Abstract

Global warming has been commonly accepted to facilitate species’ range shifts across latitudes. Cross‐latitudinal transplantations support this; many tree species can well adapt to new geographical areas. However, these studies fail to capture species’ adaptations to new light environment because the experiments were not designed to explicitly separate species’ responses to light and temperature. Here we tested reaction norms of tree seedlings in reciprocal transplantations 1,000 km apart from each other at two latitudes (60°N and 69°N). In contrast to past studies, we exposed our experimental plants to same temperature in both sites (temperature of 60°N growing site is recorded to adjust temperature of 69°N site in real time via Internet connection) while light environment (photoperiod, light quality) remained ambient. Shoot elongation and autumn coloration were studied in seedlings of two deciduous trees (*Betula pendula* and *Sorbus aucuparia*), which were expected to respond differently to day length. *Sorbus* as a member of Rosaceae family was assumed to be indifferent to photoperiod, while *Betula* responds strongly to day length. We hypothesized that (1) southern and northern populations of both species perform differently; (2) southern populations perform better in both sites; (3) autumn phenology of southern populations may delay in the northern site; (4) and *Sorbus aucuparia* is less dependent on light environment. According to the hypotheses, shoot elongation of northern population was inherently low in both species. An evolutionary consequence of this may be a competitive success of southern populations under warming climate. Southern population of *B. pendula* was delayed in autumn coloration, but not in growth cessation. *Sorbus aucuparia* was less responsive to light environment. The results suggest that light provides selection pressure in range shifts, but the response is species dependent.

## 1 INTRODUCTION

Temperature zonation is a key factor behind species geographical distribution (Dahl, 1951; Hutchins, 1947). Global warming scenarios predict 4–7°C increase in temperature for arctic areas, and even 12°C for winter time (ACIA, 2005; IPCC, 2014). The warming climate may affect plant performance as differences in adaptations to local climate may be less marked in warmer conditions (Chuine, 2000). However, one significant impact of warming climate is species and populations range shifts, even in a range up to 1,000 km poleward (ACIA, 2005). Vegetation from boreal zone may thus displace subarctic and arctic vegetation.

Species’ range shifts have been investigated quite intensively during recent years. Limiting the Scopus database literature search to concern only vegetation, the head words “climate warming” and “species’ range shifts” together result in almost 70 hits during the past 5‐year period (2011–2016). However, less attention is paid on the effects of arctic light environment (i.e., photoperiod, spectral composition or light intensity) on plants under northward range shifts (Markkola, Saravesi, Aikio, Taulavuori, & Taulavuori, 2016; Taulavuori, 2013; Taulavuori, Taulavuori, Niinimaa, & Laine, 2010), although importance of photoperiod is well‐recognized in this context (Saikkonen et al., 2012; Savolainen, Pyhäjärvi, & Knürr, 2007). For example, spectral composition may affect elongation of trees and thereby reduce competitive ability for light. Removal of blue light (400–500 nm) from light spectrum has demonstrated that elongation may be regulated through changes in light quality (Sarala, Taulavuori, Karhu, Laine, & Taulavuori, 2011; Taulavuori et al., 2005). Blue wavelengths are in essential role in range shift issues, as their relative proportion is high during night and evening hours of polar summer (Taulavuori, Taulavuori, et al., 2010).

Adaptations to light environment is particularly important for species having wide geographic distribution range and limited dispersion capacity. This is typical for trees, which exhibit usually high genetic variation and thereby they are adapted to local environments (Hereford, 2009; O'Neill, Hamann, & Wang, 2008; Savolainen et al., 2007). Correct timing of growth cessation is one of the most important adaptive traits in northern environments. Cessation of apical shoot elongation of northern trees is the primary event leading to further preparation for winter dormancy period (Junttila, 2007). Photoperiod is the major environmental factor initiating the cease of elongation in species with free growth pattern, that is, most deciduous trees. Critical photoperiod is the shortest day length prior to cease in elongation, and it increases across latitudes indicating that trees have local adaptations to photoperiod. In controlled indoor experiments, critical photoperiod is independent of temperatures (Junttila, 2007), while experiments conducted in the field have indicated correlation between photoperiod (night length) and temperature (Hänninen, 2016; and references therein). Usually, tree genotypes transplanted too far southward suffer from early growth cessation, and those transplanted too far northward are susceptible to delay in frost hardening (Evans et al., 2016; Taulavuori, 2013), although some studies have not observed the delayed frost hardening process (Taulavuori, Sarala, & Taulavuori, 2010).

**Figure 1:**
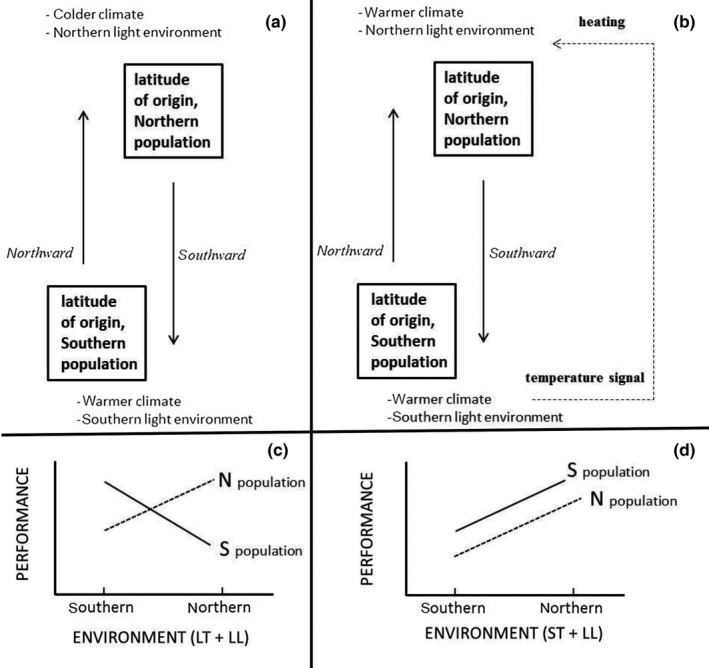
Common garden experiment and its modification for the study: (a) Schematic presentation of the conventional transplantations in common garden experiment; (b) design of the presented study, modified from the conventional common garden experiment; (c) expected reaction norms of populations under conventional common garden experiment, where best performance occurs in locally adapted environment (LL, local light environment; LT, local temperature) (modified from Savolainen et al., 2007); (d) One example of the possible hypotheses of the study: Southern population performs better than northern population in both sites, for example, benefits from longer growing season in the north (LL, local light environment; ST, southern temperature) (modified from Taulavuori, 2013)

Plant adaptations to local versus changed environment have been studied in common garden experiments, where plants originating from high and low latitude populations are reciprocally transplanted to south and north. Despite these common garden experiments provide valuable information both on theoretical and on practical perspectives, such experiments per se cannot be used to predict outcomes of plant competition under range shifts caused by warming climate. Figure 1a illustrates that simple northward transplantation is insufficient from the point of view of range shifts, as southern vegetation falls into climate colder than they are locally adapted to. Prerequisite for species range shifts is warmer climate in high latitudes where light environment is different. To our knowledge, this is the first study that applies this concept in an experimental approach with reciprocal transplantations in equal temperatures (Figure 1b).

Our objective is to study experimentally the effect of subarctic light environment on performance of southern and northern populations of tree seedlings in elevated temperatures mimicking warming climate. We investigated this issue with two species, *Betula pendula* and *Sorbus aucuparia*, as both belong to light demanding species (Giertych, Karolewski, & Oleksyn, 2015). *Sorbus aucuparia* is also an interesting species, as its autumn phenology may not be light dependent (Heide, 2011). It is hypothesized that: (1) Southern and northern populations perform differently in the subarctic light environment. (2) Southern population performs better than northern population in both sites and benefits from longer growing season due to longer days and elevated temperature. (3) On the other hand, northern light environment may delay autumn phenology of southern tree seedlings in the northern light environment. (4) *Sorbus aucuparia* is less dependent than *B. pendula* on the light environment.

## 2 MATERIALS AND METHODS

**Figure 2:**
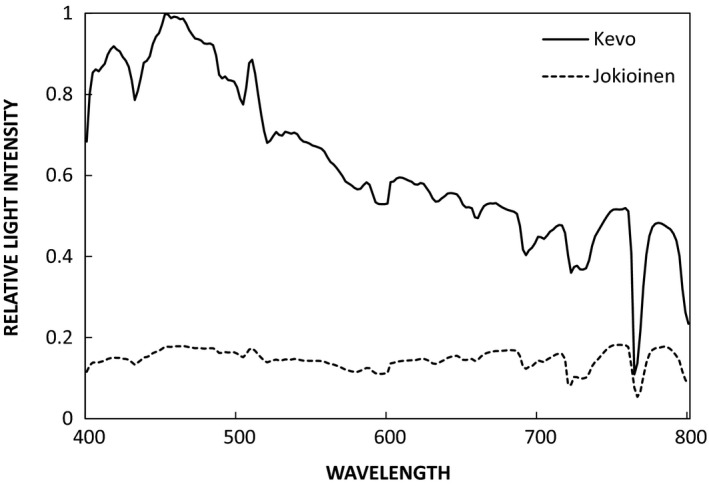
Difference in light spectra in experimental sites at 60°N (Jokioinen) and 69°N (Kevo) latitudes, measured around summer solstice at 10.00 p.m. Blue‐to‐red (445–455 vs. 655–665 nm) ratio in Jokioinen and Kevo were 1.15 and 1.91, respectively, indicating bluer light environment during evening and night of polar summer. Light spectra were measured using OL 754 Optronic Laboratories

Simulated southern climate (SSC) experiment was established to study reaction norms of southern and northern populations of silver birch (*B. pendula*) and rowan (*S. aucuparia*) in southern (Jokioinen, 60.48ºN; 23.10ºE) and northern (Kevo, 69.45ºN; 27.00ºE) light environments in the field. The experiment simulated populations’ range shifts under warming climate (Markkola et al., 2016; Taulavuori, 2013). The experiment is based on the “transfer of the southern temperature climate toward north” with plexiglass chambers equipped with electric heating in the north. Southern temperature was the driving signal for heating in the north. No heating was applied in chambers in the southern site. The northern and southern experimental sites used in the present experiment differ from each other significantly due to light environment. The two most conspicuous differences related to light environment are photoperiod and light quality. Day length varies seasonally between summer and winter for 14 and 24 hr in southern and northern sites, respectively. In addition, blue light is proportionally high during night/evening hours of polar summer without sunset (Taulavuori, Taulavuori, et al., 2010). Figure 2 illustrates this difference of the light spectra of present study sites in Kevo (northern site) and Jokioinen (southern site). Moreover, cloudy weather increases the amount of blue light in the spectrum (Lee & Hernández‐Andrés, 2005).

### 2.1 Experimental setup

**Figure 3:**
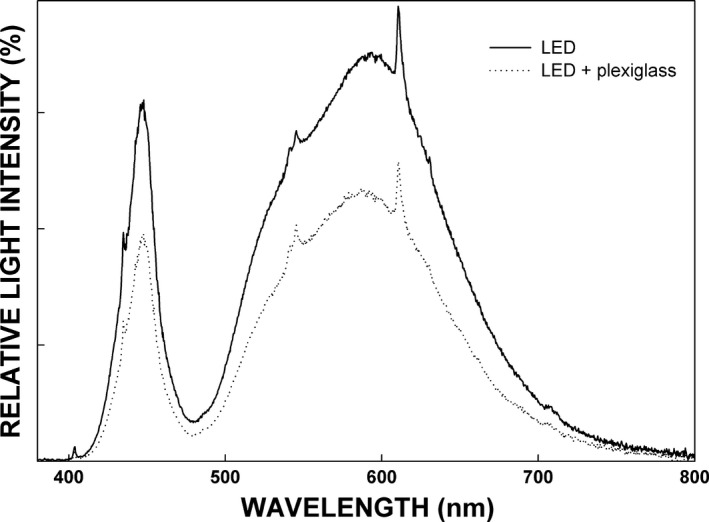
Example of effect of used plexiglass on light properties. Light intensity (PAR) diminished approx. 25%, but no changes could be observed in spectral composition (light quality). The spectra are laboratory measurements made at 540 μmol m^−2^ s^−1^ PAR (Licor 6400 sensor) provided by Valopää LED light (VP2503). Light spectra were measured using Ocean Optics USB2000 + RAD spectroradiometer

Ten plexiglass chambers (i.e., mini‐greenhouses, size of 1 × 1×1 m) were established in both experimental sites. The chambers were made of 16 mm thick polycarbonate cell plates (Es‐An Ltd , Kalanti, Finland). These plates were used as walls and roof of the chambers because of their good insulation capacity against temperature changes and relatively good transparency for light without changes in spectral composition (Figure 3). The plates were assembled in aluminum frame.

The chamber prototype with heating system was developed and tested in winter 2010 as a graduate work in Oulu Applied University (Niskakangas, 2010). Highest temperature difference in January (i.e., the coldest month) between Jokioinen and Kevo was ∆ 18.3°C, calculated from long‐term data (Drebs, Nordlund, Karlsson, Helminen, & Rissanen, 2002). Based on this difference, an energy demand (425 W) for heating system was further calculated. Therefore, chamber unit was equipped with two sets of 10‐m‐long DEVI pipeguard‐25 floor heating cable having heating capacity of 25 Wm^2^. The cables were mounted inside the chamber, around the frame with no covering on the walls. An air circulating fan (Onninen Inwall 6/15) was installed to equalize the temperature difference between corners and middle area. Soil inside the chambers was insulated against possibility of surrounding ground frost by 50 mm styrofoam plates (Finfoam) to the depth of 0.4 m. Insulating capacity of Finfoam is excellent as the heat conductivity is only 0.035 Wm^−2^ K^−1^.

The chamber experiment was established during 2011–2013 with test runs during winter 2012–2013. The information logistics link the chambers in southern (Jokioinen) and northern (Kevo) sites; the chamber temperature is recorded in Jokioinen and adjusted the same in Kevo through a real‐time Internet connection. This experimental setting allows us to explicitly separate the responses of experimental seedlings to light and temperature. In practice, this means equal temperature (i.e., southern) and latitudinal local light environment in both sites. Temperature measurement, set, and control were carried out using automation developed by Ouman Ltd, a company specialized in intelligent heating systems. A TMO/NTC 10 outdoor sensor was assembled inside one chamber in Jokioinen (60°N) to measure temperature, which was sent online via EH‐NET Internet (3G) server to Kevo (69°N) to set temperature for chambers there. Similar sensor recorded also the ambient and chamber temperatures in Kevo. Heating regulator (EH‐868 version 2.4.8.) used the temperature signal from Jokioinen to set and control the relays (RY1) guiding the function of heating cables of each chamber in Kevo. TEU‐LU (PD Produal) temperature sensors inside each chamber transmitted the temperatures to be saved in server in 10‐min intervals.

Warming succeeded well during the actual experiment in growing season 2015, which resulted in similar accumulation of temperature sum (approx. 1,100 degree days, > +5°C threshold) in both sites (Figure 5). Temperature elevation in Kevo chambers was approx. 7°C higher than long‐term ambient averages there. This is in line with climate change scenarios for arctic temperatures and subsequent species’ range shifts (ACIA, 2005).

### 2.2 Plant material

We selected silver birch (*B. pendula*) and rowan (*S. aucuparia*) as model species. These species were studied because of their demand for light (Giertych et al., 2015) and reported differences in response to photoperiod: Autumn phenology of silver birch populations is dependent on local day length (Viherä‐Aarnio, Häkkinen, Partanen, Luomajoki, & Koski, 2005) while rowan is less dependent of photoperiod (Heide, 2011). Seedlings originating from southern and northern populations were used in the experiment. We acknowledge that responses of mature trees and tree seedlings may differ from each other. Tree seedling stages are sensitive to cold temperatures (Renard, Mcintire, & Fajardo, 2016), and slow growth rate during early years of northern populations could promote survival under snow cover in winter for some years longer. On the other hand, growth rate during seedling stage is an important determinant in competition between local and invasive populations and species in the north.

Seed material was collected from wild plants originated from naturally grown local maternal trees, that is, not from silviculturally produced seed orleftds. Silver birch seeds from >15 maternal trees per latitude were collected and provided by commercial seed producing companies, and rowan seeds from >15 maternal trees per latitude were collected by research team members (Table 1). Seedlings were produced from seeds at the Botanical Gardens, University of Oulu in spring 2015. Seeds were stratified in cold (5°C) 1–3 months prior to sowing in March 2015. Seedlings were grown in individual pots in nonfertilized peat in greenhouse 2.5 months before starting the experiment.

**Table 1:** Studied tree species and populations, and latitude and collection site of the seed material with supplier of the seed source

Species	Population	Latitude (°N)	Seed collection site	Supplier
*Betula pendula*	Southern	61.3	Hartola	Metsätapio Ltd
	Northern	67.4	Kittilä	Siemenforelia Ltd
*Sorbus aucuparia*	Southern	60.5	Anjalankoski	Research group
	Northern	67.4	Kittilä	Research group

Seedlings were planted in 3‐L plastic pots in late May 2015. Potting soil was prepared by mixing sand and nonfertilized, acidic peat (Kekkilä F6) in 1:2 v/v. Piece of landscape fabric was inserted on the bottom of each pot to prevent root growth outside the container. Each seedling was given 50 mL of raw forest humus as mycorrhizal fungal source. Humus was collected in May 2015 from a mid‐latitude (Kälviä, 63.4°N) mature coniferous forest which harbored both the studied tree species. Humus was homogenized and mixed before adding it into planting hole with seedling.

Seedlings were transferred to experimental chambers in Kevo (69°N) and Jokionen (60.5°N) in 25–29 May 2015. Four individuals from southern and northern origin of each species were set in each chamber (i.e., total number of seedlings/chamber is: 4 individuals × 2 species × 2 origins = 16 seedlings) in random order. A similar set of *Pinus sylvestris* and *Picea abies* seedlings were also planted in individual pots into same experiment, making total number of 32 seedlings in each chamber (Appendix S1). Pots were embedded in sandy soil inside the chambers to prevent warming of root systems. Seedlings were watered on daily–weekly basis depending on weather conditions. Roofs of the chambers were opened on warm, sunny days to prevent excessive heating inside chambers. South‐faced walls were open in the southern site throughout the growing season, and also temporally removed from chambers of the northern site during warm periods.

### 2.3 Data collection and analyses

Shoot elongation was measured four times on site during growing season 2015: (1) on transplantation days in week 22 (Kevo 25th May, Jokioinen 28th May), (2) in mid‐summer in week 28 (Kevo 7th and 8th July, Jokioinen 10th and 11th July), (3) in late‐summer in week 33 (Kevo 16th and 17th August, Jokioinen 19th August), and (4) in autumn in week 37 (Kevo 13th and 14th September, Jokioinen 16th September). Shoot elongation was measured with a ruler (±1 mm) from pot surface to the shoot top.

Evaluation of leaf coloring was carried out on site during the last elongation measurements in week 37 in September. At the same time, samples were collected for leaf reflectance measurements to be performed in laboratory (University of Oulu). Each seedling was evaluated and scored on a scale 1–3, meaning following: 1, no coloring (green); 2, approx. 50% of leaves display coloring; 3, whole seedling display coloring. Autumn coloration was evaluated by visual inspection by one person. In each analysis (i.e., visual evaluation of leaf coloring or leaf reflectance), results of four seedlings (per species and population) were averaged to represent a value for one replicate chamber.

Spectral measurements and calculated parameters were performed to obtain additional information for autumn coloring. One leaf per seedling, representing average color to eye, was collected into plastic bag prior to storing in cool (approx. +5°C) styrofoam ice box for transport in the University of Oulu, where the bags were stored in fridge for few days period of reflectance measurements. Leaf reflectances were scanned in random order over range 400–700 nm by 1‐nm intervals (OL 754 Integral Sphere, Optronic Laboratories). Calculated parameters were reflectance sums between ranges 540 and 560 nm for green and 650 and 670 nm for red colors, and 565–585 for yellow color. A narrow range 663–664 (i.e., chlorophyll's absorption peak in red area) was scanned for indication of chlorophyll (a). Finally, green‐to‐red (G:R) and green‐to‐yellow (G:Y) ratios were calculated to indicate degree of verdancy (i.e., greenness). Verdancy reduces during leaf coloring and thus decreases the ratio (Taulavuori, Pihlajaniemi, Huttunen, & Taulavuori, 2011). Indirect degree of chlorophyll was obtained through reciprocal function (*y* = 1/*x*) of corresponding reflectance. The calculation is based on the fact that chlorophyll is responsible of absorption of red light, and the reflection of red light increases as a function of decreasing chlorophyll content.

Statistical analyses were performed using IBM SPSS Statistics 22 package. Two‐way ANOVA was used to test differences between populations and light climate. Seed origin and experimental site were used as fixed factors. The species were tested independently as well as the separate sampling days in elongation. When ANOVA assumptions on normal distribution or variance homogeneity were violated, square root transformation (i.e., for the data on elongation of all measurement days except for the first) and reciprocal transformation (i.e., index for chlorophyll A in birch and G:Y ratio in both species) we used. Games‐Howell post hoc test was employed to test differences between groups in case (i.e., G:R in birch) when ANOVA assumptions could not be fulfilled. Groups used for testing were as follows: (1) southern population in southern site, (2) southern population in northern site, (3) northern population in southern site, and (4) northern population in northern site.

## 3 RESULTS

**Figure 4:**
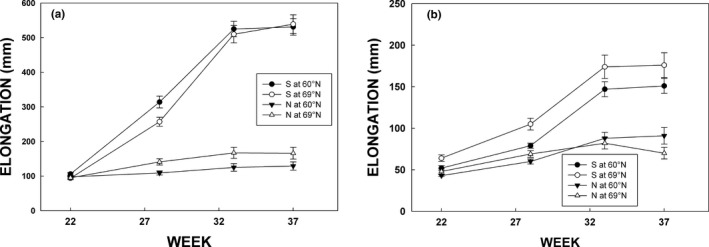
Elongation of silver birch (a) and rowan (b) during period from the beginning of June to mid‐September 2015. Circles and triangles denote for southern and northern populations, respectively, and filled and open symbols indicate southern (Jokioinen, 60°N) and northern (Kevo, 69°N) growing sites, respectively. Each measurement is a mean (±*SE*) of ten chambers (*n* = 10)

Shoot elongation in silver birch varied significantly according to population origin (Figure 4a). Elongation in northern population was minor, and statistically differed from elongation of the southern population in July, August, and September (Table 2). Significant population × experimental site interaction in week 28 in July indicated also that both populations elongated better in their latitude of origin (Figure 4a, Table 2). Similar trend continued in the northern population throughout the experiment, while the difference in elongation leveled off in the case of southern population toward the end of growing season.

**Table 2:** Results of ANOVA for shoot elongation in silver birch and rowan in different populations (northern/southern origin) and experimental site (northern 69°N/southern 60°N) during growing season (July–September)

Source of variation	*df*	MS	*F*	*p*
Silver birch				
Shoot elongation in July (week 28)				
Population	1	309.7	210.1	<.001
Site	1	0.12	0.08	.779
*P* × *S*	1	22.2	15.0	<.001
Error	34	1.5		
Shoot elongation in August (week 33)				
Population	1	1,097.3	383.6	<.001
Site	1	4.6	1.6	.212
*P* × *S*	1	10.3	3.6	.067
Error	34	2.9		
Shoot elongation in September (week 37)				
Population	1	1,164.2	356.0	<.001
Site	1	6.9	2.1	.156
*P* × *S*	1	4.5	1.4	.249
Error	34	3.3		
Rowan				
Shoot elongation in July (week 28*)*				
Population	1	21.8	42.7	<.001
Site	1	8.3	16.3	<.001
*P* × *S*	1	1.3	2.4	.127
Error	34	0.51		
Shoot elongation in August (week 33)				
Population	1	53,724.6	60.1	<.001
Site	1	1,011.6	1.1	.295
*P* × *S*	1	2,755.8	3.1	.088
Error	34	893.3		
Shoot elongation in September (week 37)				
Population	1	65,144.1	63.6	<.001
Site	1	33.6	0.03	.857
*P* × *S*	1	4,852.5	4.7	.037
Error	34	1,024.2		

**Figure 5:**
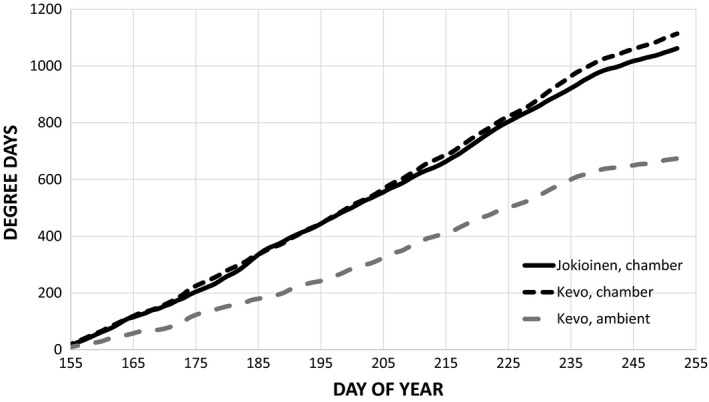
Accumulated degreed days above +5°C threshold in the chambers of Jokioinen (signal T) and Kevo (target T), and in the ambient air Kevo during growing season 2015. Note: Accumulation of dd is artificial as temperature recordings were started during transplantation of seedlings

Consistently with silver birch, the elongation of southern population of rowan was also superior compared to the elongation of northern population in July, August, and September (Figure 4b, Table 2). The elongation was generally significantly higher in the northern growing site in July (week 28), but final outcome in the elongation in week 37 in September indicated population × growing site interaction, that is, both populations elongated less in their native latitude (Table 2).

**Figure 6:**
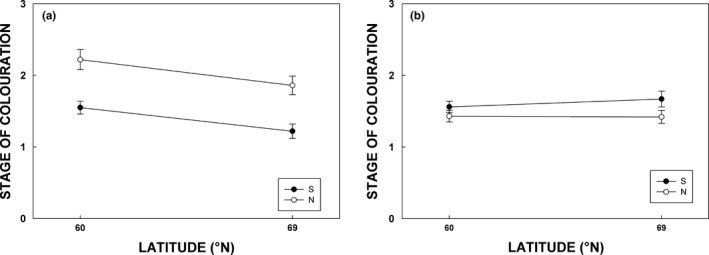
Stage in autumn coloration (visual evaluation) of silver birch (a) and rowan (b) in mid‐September (week 37) 2015. Colored and open symbols denote for southern and northern populations, respectively. Latitude in *X*‐axis indicate southern (Jokioinen, 60°N) and northern (Kevo, 69°N) growing sites, respectively. Each measurement is a mean (±*SE*) of ten chambers (*n* = 10). Stages of coloration: 1, no observable coloring; 2, approx. 50% of the leaves display coloring; 3, most of the leaves display coloring

Coloration in northern population of silver birch was advanced (i.e., higher average scores in range of 1–3) compared to southern population in both sites with approximately 50% coloration by visual observation in northern seedlings (Figure 6a, Table 3). In addition, autumn coloration was delayed in the northern growing site (Figure 6a, Table 3). Coloration of rowan showed no difference between growing sites, but southern rowan population had advanced coloring compared to northern population (Figure 6b, Table 3).

**Table 3:** Results of ANOVA for autumn coloration in silver birch and rowan in different populations (northern/southern origin) and experimental site (northern 69°N/southern 60°N)

Source of variation	*df*	MS	*F*	*p*
Autumn coloration in silver birch				
Population	1	4.0	30.9	<.001
Site	1	1.1	8.5	.006
*P* × *S*	1	0.002	0.01	.906
Error	34	0.13		
Autumn coloration in rowan				
Population	1	0.33	4.3	.047
Site	1	0.02	0.25	.618
*P* × *S*	1	0.04	0.47	.497
Error	34	0.08		

**Figure 7:**
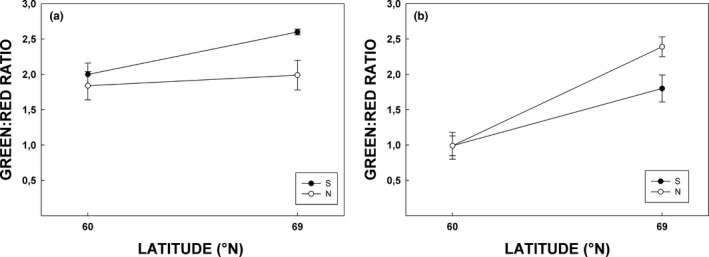
Green: red ratio (measured from leaf reflectances at 540–560 nm for green; 650–670 nm for red) of silver birch (a) and rowan (b) leaves during period from the beginning of June to mid‐September 2015. Explanations otherwise as in Figure 5

Leaf verdancy (i.e., greenness) of silver birch expressed as green‐to‐red ratio (G:R) was highest in southern population grown in the northern site (Figure 7a). It differed significantly from ratios of both populations in southern growing site (Games‐Howell: *p* = .018) and marginally (*p* = .067) from northern population in the same growing site. In rowan, significant difference in G:R was observed between growing sites pointing to delayed coloring in the northern site (Figure 7b, Table 4). There were also a marginally significant differences between rowan populations and in population × site interaction (Table 4), indicating that northern population in the north site was more delayed in coloration compared to southern population.

**Table 4:** Results of ANOVA for leaf verdancy (i.e., green:red ratio, green:yellow ratio, and chlorophyll A index) in silver birch and rowan in different populations (northern/southern origin) and experimental site (northern 69°N/southern 60°N)

Source of variation	*df*	MS	*F*	*p*
Silver birch				
Green:yellow ratio				
Population	1	0.23	4.1	.050
Site	1	0.50	8.8	.005
*P* × *S*	1	0.18	3.1	.088
Error	35	0.06		
Chlorophyll A index				
Population	1	42.5	3.9	.057
Site	1	1.6	0.15	.706
*P* × *S*	1	49.4	4.5	.041
Error	35	10.9		
Rowan				
Green:red ratio				
Population	1	0.93	3.8	.060
Site	1	10.6	43.2	<.001
*P* × *S*	1	0.88	3.6	.068
Error	32	0.25		
Green:yellow ratio				
Population	1	0.32	2.8	.104
Site	1	3.3	29.1	<.001
*P* × *S*	1	0.27	2.4	.134
Error	32	0.12		
Chlorophyll A index				
Population	1	<0.001	0.02	.880
Site	1	0.005	4.5	.042
*P* × *S*	1	0.001	1.2	.290
Error	32	0.001		

**Figure 8:**
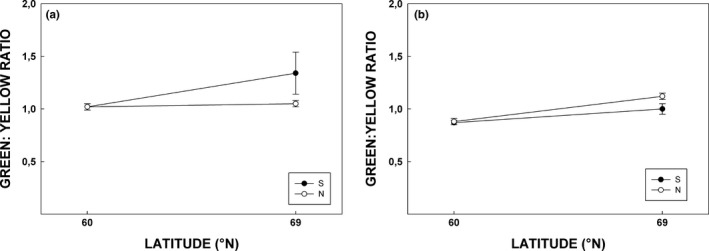
Green: yellow ratio (measured from leaf reflectances at 540–560 nm for green; 565–585 nm for yellow) of silver birch (a) and rowan (b) leaves during period from the beginning of June to mid‐September 2015. Explanations otherwise as in Figure 5

Greenness of silver birch expressed as green‐to‐yellow ratio (G:Y) was higher in northern than in southern growing site (Figure 8a, Table 4). There was also a marginal difference (i.e., *p* = .050) between birch populations indicating slightly more greenness in leaf color in southern population (Figure 8a, Table 4). Similarly, G:Y ratio in rowan was higher in the northern site, but the populations did not differ (Figure 8b, Table 4).

**Figure 9:**
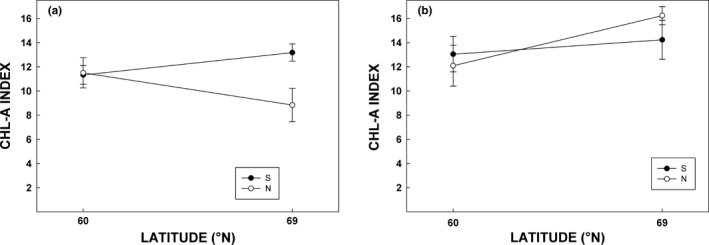
Chlorophyll A index (measured from leaf reflectances at 663–664 nm) of silver birch (a) and rowan (b) leaves during period from the beginning of June to mid‐September 2015. Explanations otherwise as in Figure 5

Index for chlorophyll A content in silver birch populations showed significant population × site interaction, indicating that in northern site chlorophyll index of both populations diverged from the southern site (Figure 9a, Table 4). The only significant difference in chlorophyll index values of rowan was between the sites, indicating higher chlorophyll contents in the north (Figure 9b, Table 4).

## 4 DISCUSSION

Reciprocal transplantation experiments across the latitudes suggest that trees are adapted to local environment (Kawecki & Ebert, 2004; Savolainen et al., 2007; and references therein). Common garden experiments locating in mid‐range of latitudinal transects, usually show best growth for local populations of the experimental sites, while at the range margins, the local adaptations may break down (Savolainen et al., 2007). According to Oleksyn, Reich, Tjoelker, and Chalupka (2001), southward transplantations usually result in growth depression, obviously related to shorter days during growing season. Inferior growth of northward transplanted southern populations may result from variable factors (Oleksyn et al., 2001). The present study, to our knowledge, reports the first experimental setup that enables investigation of reaction norms of southern and northern populations under high and low latitudinal light environment without distortion caused by temperature difference between sites. The most striking result is that shoot elongation in northern populations of both species (silver birch and rowan) was inherently low. This is in agreement with results on Scots pine seedlings indicating lower growth potential in northern populations (Matias & Jump, 2014; Reich & Oleksyn, 2008; Taulavuori, Taulavuori, et al., 2010). In addition, recent literature reports that subarctic tree populations growing at their northern margin have less variability in genes controlling phenology than in southern populations (Mimura & Aitken, 2007; Savolainen et al., 2011). Given that global warming allows range shifts of up to 1,000 km, it could be concluded that southern populations are potentially highly competitive. However, this does not exclude the possibility that southern tree populations may be more vulnerable to extreme weather events than northern populations.

In many trees with free growth pattern (e.g., silver birch), shoot elongation ceases at critical night length (CNL), which decreases clinally with increasing latitude (Viherä‐Aarnio et al., 2005). A clinal variation in growth cessation has shown in Norway spruce over latitude range from 54 to 66°N (Chen et al., 2014). In principle, southward and northward transplanted trees cease growth prematurely and in delay, respectively (Rohde, Bastien, & Boerjan, 2011). Elongation of silver birch in the present experiment is consistent with maximal fitness in local sites (Kawecki & Ebert, 2004; Savolainen et al., 2007). Unexpectedly, the elongation of the southern population was not delayed in the northern site with longer days. Previous CNL results on silver birch (Viherä‐Aarnio, Häkkinen, & Junttila, 2006) may be projected to estimate growth cessation of southern population in the northern site. And if doing so, CNL should have been met in week 32 as also observed in the present investigation. However, the northern population in the southern site elongated throughout the experiment in the photoperiod longer than CNL, that is, shortest night length in the southern site was 5 hr (vs. CNL approx. 4 hr for northern population).

Elongation of rowan appears not to be locally adapted**:** Southern population elongated better in the northern site, whereas northern population elongated better in southern site. The finding nevertheless does not support the general phenomenon of delayed growth cessation of southern populations in the north due to later occurring CNL (Rohde et al., 2011), as the elongation rate seems to be higher in the northern site throughout the summer. Indeed, there is evidence that it is temperature rather than photoperiod, which controls growth cessation in *Sorbus* species (Heide, 2011). Either southern population may have been more effective in use of the long days in the north, or it has better tolerance to northern light quality.

Hänninen (2016) reviewed so‐called joint effect model (Koski & Selkäinaho, 1982), according to which elongation ceases earlier in warm growing seasons, that is under high temperature sums. The northern populations of both species experienced higher temperature sums than where they have been adapted and elongated significantly less than southern populations. We assume that possible temperature effect would appear through acceleration of metabolic rates and should be seen as higher slope in the elongation curve and subsequently advanced lag phase. Therefore, the poor elongation of northern populations cannot be explained by temperature effects.

The different growth potential of the studied populations may be a result from survival and capacity adaptations (Hänninen, 2016; and references therein). Briefly, capacity adaptation facilitates competitive ability, and survival adaptation refers to avoidance of stress. Late spring phenology and early autumn phenology guarantee maximal survival in high latitudes without risk of freezing. In turn, early spring phenology and late autumn phenology maximize the capacity adaptation pattern, that is, maximal use of growing season. Late spring phenology (Vitasse, Lenz, & Körner, 2014; and references therein) and slow deacclimation rate (Taulavuori et al., 2004) are typical for plants at high latitudes. It is emphasized that delayed spring phenology may shorten the growing season and reduce productivity in trees of northern climate (Arora & Taulavuori, 2016). The elongation results of the present investigation fit well into the concept of survival and capacity adaptations: Northern and southern populations of studied species exhibit high survival and capacity adaptations, respectively.

Actually, final result in elongation may be affected by light quality also through other mechanisms than growth cessation. Photomorphogenic responses have also potential to control the elongation. Northern populations may require higher amount of red (R) and far red (FR) light to maintain growth than southern populations (Mølmann, Junttila, Johnsen, & Olsen, 2006). This requirement could explain the slightly better elongation of northern rowan populations in the southern site, but not other results. Mølmann et al. (2006) also observed that blue light (B) may delay bud set in any populations of Norway spruce. It is also shown in Scots pine that B may significantly reduce elongation in subarctic experiments (Sarala, Taulavuori, Taulavuori, Karhu, & Laine, 2007; Sarala et al., 2011; Taulavuori et al., 2005). Northern population of Scots pine has shown to be also more sensitive (i.e., benefit more of removal of B) than southern population to B, and removal of B from spectrum has decreased anthocyanin synthesis of many species (Sarala et al., 2011). Accordingly, Taulavuori et al. (2011) suggested high anthocyanin concentration to act as an adaptation to northern light environment. Given that the high synthesis of anthocyanins synthesis would be an adaptation to proportionally high B in the northern light environment (Figure 1), a trade‐off between growth and protection could explain the minor elongation of the northern origin, at least to some extent.

Autumn coloration in silver birch, evaluated by visual scoring, occurred as precisely as a calendar read. The related species *Betula pubescens* starts coloring (i.e., 50% yellowing as in our study) in week 37 (Poikolainen, Tolvanen, Karhu, & Kubin, 2016), exactly as did the northern populations in the present experiment**.** Coloration in southern Finland starts 2–3 weeks later (Poikolainen et al., 2016) in accordance with the delay observed in the southern population in the present study. It is suggested that latitudinal patterns in leaf coloring correlate with temperature (Doi & Takahashi, 2008). On the other hand, Gill et al. (2015) proposed that leaf senescence is sensitive to photoperiod in the high latitudes. Also other studies have shown that species in high latitudes are generally more responsive to photoperiod (Stinziano & Way, 2014; Way & Montgomery, 2015). The present investigation on silver birch coloration indicates two phenomena: (1) “southern” (i.e., 61°N) population of the experiment was responsible to photoperiod and was delayed in leaf coloring in both sites when compared to northern population; (2) leaf coloring of both populations advanced in southern site. Taking together, these findings suggest that latitudinal patterns in leaf coloring correlate with photoperiod, temperature, and genome, at least from 61°N northward.

Measurements on leaf verdancy give support to the result obtained by visual scoring. Green:red ratio of silver birch leaves is around 2.5 during luxuriant growth, which decreases to levels 2–1 during coloration of warm and cold autumns, respectively (Taulavuori et al., 2011). Consistently, leaves of southern silver birch population indicated no coloration in the northern site and had G:R ratio 2.5, and those which had started to color had G:R ratio 2 or below. No comparable reference could be found for verdancy expressed as either green:yellow ratio or chlorophyll index as, but both confirm the above findings: Southern silver birch population in the north was delayed in autumn coloring.

Leaf coloring in rowan varied less than coloring of silver birch, and the degree of coloring was generally delayed compared to silver birch. Visual scoring indicated that the southern population of rowan was slightly advanced in coloring. All the other coloring‐related variables, except for visual scoring, speak for the delayed coloring in the northern site. Rather advanced than delayed coloration of southern population is in accordance with the lacking potoperiodic response in this species (Heide, 2011). Also generally delayed coloration in the northern site could contradict with this interpretation, as the temperature was equal under in both site. Therefore, one explanation for different coloration rate could be spectral composition, in agreement with other publications (Junttila, 2007; Mølmann et al., 2006).

In conclusion, a significant difference was observed in both silver birch and rowan between elongation of southern and northern populations. As we hypothesized, southern and northern populations perform differently in the subarctic light environment (Hypothesis 1). We further expected (Hypothesis 2) that southern populations of both species performed better than northern population in both sites. In accordance, performance of southern population in the northern light environment under equal temperature sums reveals high capacity adaptation traits, whereas northern populations display traits of survival adaptation. Autumn coloration of silver birch is an additional evidence for the investment of growth rather than survival in the northern light environment as it delayed in autumn coloring. Our third assumption (Hypothesis 3) was that autumn phenology of southern population delays in the north. However, no delayed growth cessation was observed in southern populations. For studies in the future, it would be interesting to follow the development of frost hardiness, which tells whether the strategy of southern population results in too high risks. Instead of that, in accordance with Hypothesis 4, rowan shows less response to light environment as expected (Heide, 2011), as it rather hastened than delayed the autumn coloration. Overall, this experiment shows that light provides selection pressure for adaptation in range shifts, but the response is species dependent.

## CONFLICT OF INTEREST

None declared.
