# Supplementary material for: Competitive success of southern populations of Betula pendula and Sorbus aucuparia under simulated southern climate experiment in the subarctic
Source: Ecol Evol. 2017 May 30;7(12):4507–17. doi: 10.1002/ece3.3026 (PMC5478116; doi:10.1002/ece3.3026)
Supplement: Supplementary file 1 [file ECE3-7-4507-s001.docx]

**Appendix S1:** Outline of the experimental setup and seedling origin in Simulated Southern Climate (SSC) experiment.


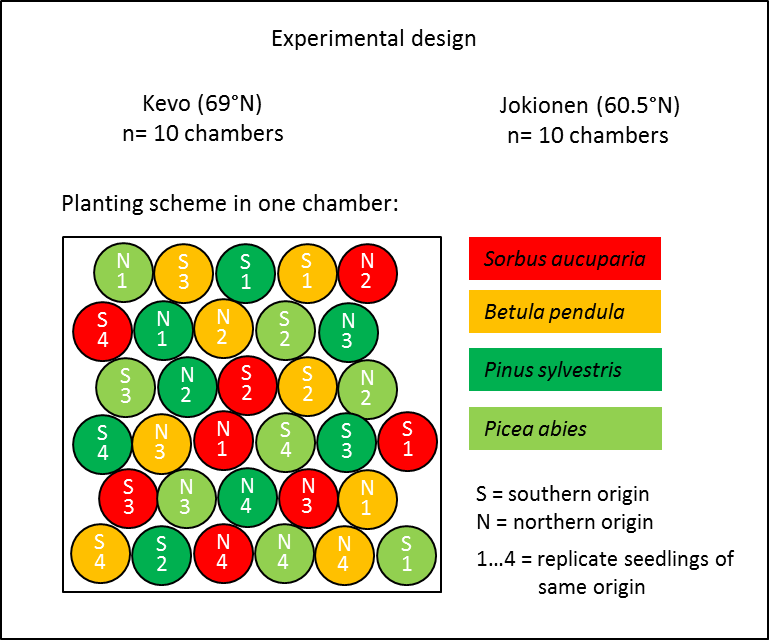


| **Species** | **Origin** | **Latitude (°N)** | **Seed/seedling origin** | **Supplier** |
| --- | --- | --- | --- | --- |
| *Sorbus aucuparia* | Southern | 60.5 | Anjalankoski | Group |
|  | Northern | 67.4 | Kittilä | Group |
|  |  |  |  |  |
| *Betula pendula* | Southern | 61.3 | Hartola | Tapio Ldt |
|  | Northern | 67.4 | Kittilä | Siemen Forelia Ltd |
|  |  |  |  |  |
| *Pinus sylvestris* | Southern | 61.1 | Eurajoki | Tapio Ldt |
|  | Northern | 67.4 | Kittilä | Siemen Forelia Ltd |
|  |  |  |  |  |
| *Picea abies* | Southern | 62.3 | Heinävesi | Pohjan Taimi Ltd, Juuka |
|  | Northern | 67.4 | Kittilä | Pohjan Taimi Ltd, Kemijärvi |
